# kRadar++: Coarse-to-Fine FMCW Scanning Radar Localisation

**DOI:** 10.3390/s20216002

**Published:** 2020-10-22

**Authors:** Daniele De Martini, Matthew Gadd, Paul Newman

**Affiliations:** Department of Engineering Science, Oxford Robotics Institute, University of Oxford, Oxford OX1 3PJ, UK; pnewman@robots.ox.ac.uk

**Keywords:** radar, mapping, localisation, place recognition, autonomous vehicles, deep learning

## Abstract

**Simple Summary:**

This paper presents a hierarchical approach to place recognition and pose refinement for Frequency-Modulated Continuous-Wave (FMCW) scanning radar localisation.

**Abstract:**

This paper presents a novel two-stage system which integrates topological localisation candidates from a radar-only place recognition system with precise pose estimation using spectral landmark-based techniques. We prove that the—recently available—seminal radar place recognition (RPR) and scan matching sub-systems are complementary in a style reminiscent of the mapping and localisation systems underpinning visual teach-and-repeat (VTR) systems which have been exhibited robustly in the last decade. Offline experiments are conducted on the most extensive radar-focused urban autonomy dataset available to the community with performance comparing favourably with and even rivalling alternative state-of-the-art radar localisation systems. Specifically, we show the long-term durability of the approach and of the sensing technology itself to autonomous navigation. We suggest a range of sensible methods of tuning the system, all of which are suitable for online operation. For both tuning regimes, we achieve, over the course of a month of localisation trials against a single static map, high recalls at high precision, and much reduced variance in erroneous metric pose estimation. As such, this work is a necessary first step towards a radar teach-and-repeat (RTR) system and the enablement of autonomy across extreme changes in appearance or inclement conditions.

## 1. Introduction

The ability to localise in an already visited environment is paramount for robotic autonomy, since it enables long-term operation. A common solution to this operational paradigm is the creation of a map as a digital representation of the traversed environment by the autonomous vehicle (AV). Depending on the detail and the organisation of the information in the map, various representations have been proposed in the last decades, including: metric, topological, or hybrid topometric ones. However, meaningful robotic tasks require only locally accurate “metric” localisation in the form of a rigid-body pose with respect to a prior map [1]. The AV will then be able to use feature matching during a live pass in the same locations to localise itself onto the map and inform its motion control.

Consider these feature-based techniques and the criticality of safety in these scenarios. In order for AVs to travel safely at higher speeds or operate in wide-open spaces where there is a dearth of distinct features, the highest level of robust sensing is required. Frequency-Modulated Continuous Wave (FMCW) radar satisfies these requirements, thriving in all environmental conditions (rain, snow, dust, fog, or direct sunlight), providing a 360° view of the scene, and detecting targets at ranges of up to hundreds of metres with centimetre-scale precision.

**Figure 1:**
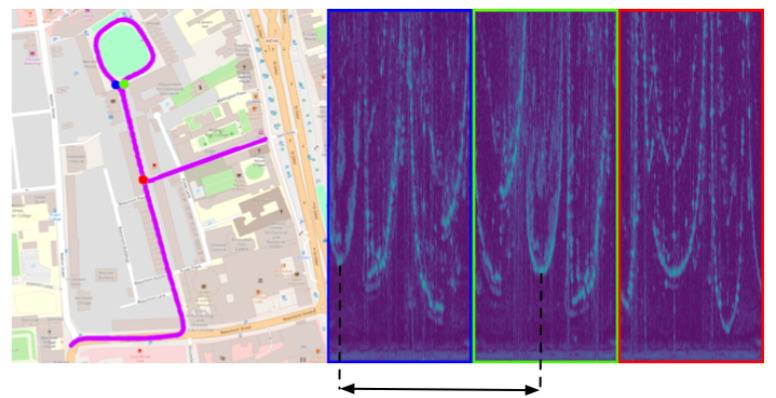
Our two-step system relies on a radar place recognition (RPR) module and a downstream pose refinement module. Firstly, consider the RPR module. Given an online query radar scan (blue dot on map and blue-framed radar image), the aim is to retrieve a correct match (green), disregarding the incorrect, although similar, radar scan the map also represents (red) and despite the obvious rotational offset. Secondly, consider the pose refinement module. Here, the rotational offset should not be disregarded and must be recovered. The range-azimuth region marked as interesting between the blue and green scans (dashed lines) is separated (solid lines) by such a rotational offset (significant wrapping around the horizontal axis), as well as a small translational offset (some movement down the vertical axis). Here, the conflict between the requirement that the RPR module be robust to small rotational and translational viewpoint differences and the requirement that the pose refinement module be precise in the same considerations are shown in this paper to be compatible in a framework that ultimately leads to a system which is more precise than RPR alone and more expedient than pose refinement alone.

Indeed, there is a burgeoning interest in exploiting FMCW radar to enable robust mobile autonomy, including ego-motion estimation [2,3,4,5,6,7], localisation [7,8,9,10,11], Simultaneous Localisation and Mapping (SLAM) [12], and scene understanding [13,14,15]. However, despite radar’s promise to deliver such capabilities, the study of these tasks is only mature for cameras and Light Detection and Rangings (LiDARs), and relatively little attention has been paid to radar for the same application. Figure 1 shows an example of application, where a scan belonging to the current, live pass (blue) is used to localise onto the previously-created map, to which a good match (green) and bad match (red) belong. The system should not only be able to identify (despite opposing viewpoints) the right match (green) but also to infer the displacement (translational and rotational) between the latter and the current position. We define this approach as a hierarchical localisation pipeline in which a rotationally-invariant radar place recognition (RPR) submodule first serves as a coarse localiser to retrieve relevant reference frames and a pose refinement step (precise in both translation and rotation) is secondly applied to the query frame and the portion of the map corresponding to the retrieved frames to estimate the metric pose.

This paper’s contribution is the proposal of a solution for such problem and can be summarised as the consolidation of two recent lines of investigation in centimetre-scale motion estimation and large-scale place recognition using FMCW radar. We adopt the vocabulary of “large-scale” from localisation literature, such as Reference [16], which are evaluated on similarly extensive urban datasets up to tens of kilometres rather than the vocabulary of works, such as Reference [1], which are benchmarked on data taken over areas as extensive as hundreds of kilometres. We view this contribution as the enabling methodology for future systems which will rely on FMCW radar as the primary sensor for long-term autonomy—for example, in an anticipated RTR application.

The remainder of this work is structured as follows. Section 2 explores related literature. Section 3 provides the prerequisite detail for the works that we build upon and how they are consolidated in the proposed system in Section 4. Section 5 provides necessary details for implementation, evaluation, and our dataset, and Section 6 and Section 7 showcase the system in that experimental setting. Section 8 compares the solution presented here against a comparable solution from the literature. Section 9 and Section 10  discuss the significance of the contribution and suggest further avenues for investigation.

The preliminary detail on RPR and radar pose refinement covered in Section 3 is from our earlier publications [2,8], while the marriage of these two systems in Section 4 is the principal contribution of this paper, along with the entirety of the experimental programme (c.f. Section 5) and experimental evidence (c.f. Section 6 and Section 7).

## 2. Related Work

This section places the contribution in the literature on radar-based odometry and localisation, experience-based navigation, and two-stage topological and metric localisation using onboard sensors.

The system we propose in Section 4 has its behaviour explored in Section 6 and Section 7 and is compared in Section 8 against some of the solutions discussed here.

### 2.1. Vision- and LiDAR-Based Lifelong Navigation

In this work, we defer to the evidence provided in the recent work in Reference [2,6,11] in proving the radars perform favourably to cameras and LiDARs in motion estimation and localisation and instead focus our experimental programme on what the proposed system *radar-only* brings to mobile robotic navigation capabilities.

However, works, such as Reference [17,18,19], demonstrate the power of the teach-and-repeat (TR) operational paradigm in enabling autonomy at scale using stereoscopic and monocular vision, as well as LiDAR. From amongst several approaches, TR has distinguished itself as being an effective solution in a number of domains, from urban autonomous driving [20] to Arctic exploration [17]. In this framework, a robot is driven along a desired path in a teach phase, during which it builds a topometric map [21,22] of the world by estimating its relative pose between consecutive keyframes. During a repeat run, localisation is performed by matching features between the live feed and those of the map, estimating the position of the robot relative to the route previously traversed.

The Experience-based Navigation (EBN) framework [20,23] for lifelong navigation builds on from TR systems by investigating the utility of experience to localisation capability over many repeat passes of a changing world.

### 2.2. Radar-Based Mapping and Localisation

Besides its superior range and despite its lower spatial resolution, Millimetre-Wave (MMW) radar often overcomes the shortcomings of laser, monocular, or stereo vision because it can operate successfully in dust, fog, blizzards, and poorly lit scenes [24]. In Reference [25], it is shown in the context of a SLAM system that while producing slightly less accurate maps than LiDARs, radars are capable of capturing details, such as corners and small walls.

Some mapping and localisation systems using similar—but not identical—radars as that used in this work are described in Reference [26,27].

The supervised learning framework presented in Reference [8,9] and which our work extends uses rotationally-invariant feature extraction and triplet-mining but does not solve for the rigid-body pose of the sensor. The cross-modal radar-satellite works presented in Reference [10,28] do solve for the metric pose. The work presented in this paper, in contrast, does not rely on external services (i.e., satellite imagery) and is presented as a radar-only solution.

In Reference [7], a method is presented for learning to predict robust keypoints for odometry estimation and metric localisation in radar. Our work is distinct in that the topological localisation aspects are trained for precisely that task—not being trained for odometry estimation and then used for localisation (having no specific loss for localisation). Additionally, our metric pose refinement is not differentiable and thus not learned end-to-end.

FMCW dopper-enabled radars have been shown in Reference [29] to be beneficial. In this work, however, we consider range-only radar.

### 2.3. Hierarchical Localisation

Often underpinning the software stack for these systems is a hierarchical localisation pipeline in which visual place recognition (VPR) first serves as a coarse localiser to retrieve relevant reference frames and a pose refinement step is secondly applied to the query frame and the portion of the map corresponding to the retrieved frames to estimate the metric pose. Recent examples in the visual domain include Reference [30,31,32,33].

The work in Reference [34] is related to ours in the sense that it can also be seen as a hierarchical approach to localisation, in the particular case of LiDAR. We see two major distinctions between Reference [34] and our work. Firstly, to achieve rotational invariance we account for the cylindrical nature of the scan formation process in our architecture, while Reference [34] maintains the rotation information and exploits it to extract the yaw displacement from the comparison of the embeddings of two scans. Secondly, in Reference [34], the measure of overlap for a pair of scans is a nonlinear, complex function that takes both embeddings as input rather than a simple distance measure. As such, the query procedure prevents the direct use of efficient searching procedures, such as the k-D-trees we use in our RPR module.

Similarly, we view the transplant of the LiDAR method developed in Reference [35] to the radar domain as in Reference [11] as a hierarchical process. In contrast to this approach, the work presented in this paper uses a *learned* embedding for RPR as opposed to a mean-reduced ring-key and recovers the full three degree-of-freedom (3DoF) pose of the vehicle as opposed to rotation-alignment alone.

## 3. Preliminaries

**Figure 2:**
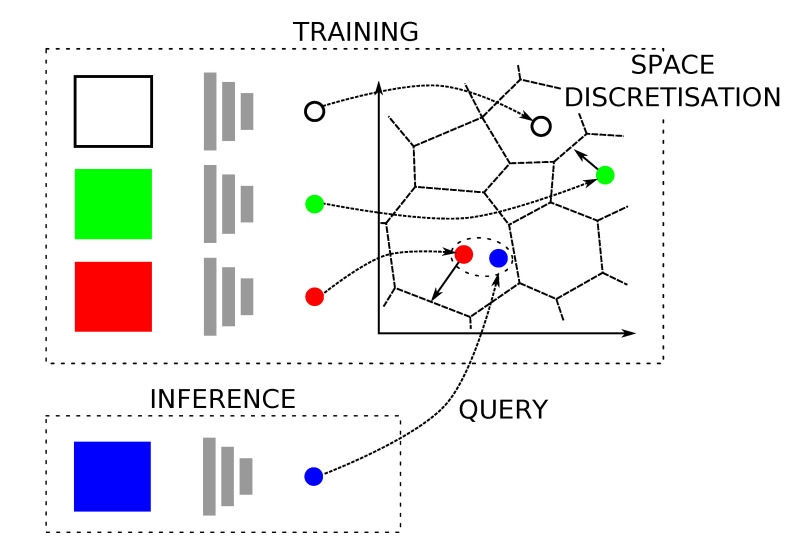
The Frequency-Modulated Continuous Wave (FMCW) radar place recognition (RPR) pipeline, from Reference [8]. The offline stages of the pipeline involve *enforcing* and *discretising* the metric space, while the online stages involve *inference* to represent the place the robot currently finds itself within in terms of the learned knowledge and *querying* the discretised space, in this case depicted using a Voronoi-like structure, which encodes the trajectory of the robot.

This section briefly describes, in Section 3.1 and Section 3.2, the two enabling subsystems which are essential to the proposed integration of Section 4. Section 3.1 describes the place recognition submodule, which is indepently illustrated in Figure 2. Section 3.2 describes the pose estimation submodule, which is based on an odometry estimation pipeline.

### 3.1. Radar Place Recognition (RPR)

Recent advances in radar-only place recognition capabilities allow us to use the FMCW sensor in lieu of global methods with other sensors, such as Global Positioning System (GPS). Here, we summarise the most salient features of the methodology, shown in Figure 2; the interested reader is referred to Reference [8] for more detail, as well as the experimental evidence of the robustness of this RPR module.

**Feature extraction:** To learn filters and cluster centres which help distinguish polar radar images for place recognition we use NetVLAD [36] with VGG-16 [37] as a front-end feature extractor—both popularly applied to the place-recognition problem. Importantly, the original implementation of the feature extractor is altered to obtain invariance to the orientation of input radar scans, including: circular padding [38], anti-aliasing blurring [39], and azimuth-wise max-pooling.

Circular padding along the azimuth direction has been applied to each convolutional layer of the VGG architecture. This is done as the polar representation of the assembled Fast Fourier Transform (FFT) returns has no true image boundary along the azimuth axis. The operation brings equivariance to rotation to the feature extraction architecture along the boundary region of the radar scan.

Downsample operations lead to high-frequency components of the signal, in this case the radar image, to cause aliasing in the sampled one. For this reason, we apply a Gaussian blur before each downsampling operation in order to reduce this effect and help the natural equivariance of the convolutional operations.

Lastly, we applied a max-pooling operation along the azimuth dimension at the end of the feature extractor. Indeed, so far the architecture has only been equivariant to rotation, meaning that a rotation of the radar scan would correspond to a rotation on the output features. Applying a max-pooling operation, which is inherently invariant to the order of the inputs, would indeed bring this invariance to the architecture itself on the dimension of application, in this case the azimuth one.

**Triplet mining:** To enforce the metric space, we perform online triplet mining and apply the triplet loss described in Reference [40].

The triplet loss can be described using the Euclidean distance function

(1) $$L\left(a,p,n\right)=\max(\left|\right|f\left(a\right)-f\left(p\right){\left|\right|}^{2}-\left|\right|f\left(a\right)-f\left(n\right){\left|\right|}^{2}+\alpha ,0),$$

where *a*, *p*, and *n* are an anchor radar scan, positive example from the same location, and negative example from a dissimilar location, respectively, and *f* represents the encoding done by the Convolutional Neural Network (CNN) to represent these scans as “embeddings”. Examples of these are shown in Figure 1 (blue, green, and red, respectively). Loop closure labels are taken from a groundtruth dataset (c.f. Section 5). Batches are constructed such that there is no overlap of the radar sensing horizon between a candidate radar scan and any anchor scan already sampled for the batch.

**Online querying:** Embedding distance is calculated as in Equation (1) above. For example, the embedding distance between a candidate in the map, $c_{i}$ and the query scan, *q*, is given by

(2) $$d\left(q,m_{i}\right)=\left|\right|f\left(q\right)-f\left(c_{i}\right){\left|\right|}^{2}.$$

In the deployed subsystem, whenever a new radar scan is available, it is encoded in the multidimensional space by performing inference upon the trained network. As will be discussed in Section 5.3, a nearest neighbour (NN) search retrieves the *n* closest neighbours, instead of a ball search out to a maximum embedding distance threshold, which has no guarantee on the number of candidates passed to the downstream pose refinement (c.f. Section 3.2). Therefore, the maximum number of queried neighbours is a design parameter and is set in Section 6 by taking into consideration the speed of the pose refinement algorithm (c.f. Section 3.2) which will be executed on pairs of radar scans, formed by the query scan and each of the retrieved neighbours. In Section 6, we show a range of recalls at high precision (c.f. Section 5.2) that are available when performing online queries which are strictly possible with reasonable compute requirements (c.f. Section 5.3).

**Training details:** Due to memory limitations on our graphical compute hardware, we crop the last 168 range bins and scale the width by a factor of 8 such that the original 400×3768 polar radar scans are input to the network with resolution 400×450. When finetuning either the original architecture or our proposed modified architecture, we initialise internal weights with the publicly available checkpoint vd16_pitts30k_conv5_3_vlad_preL2_intra_white, corresponding to the best performing model described in Reference [41], which produces embedding vectors of length 4096. As the azimuth axis remains unscaled, this does not affect rotational invariance. Our embedding triplet margin is set to 1.0 and our learning rate schedule applies a linear decay function initialised at $1\times 10^{-4}$ and settling to $5\times 10^{-6}$ at 5000 steps [42]. We terminate learning at 500,000 steps in all cases. We use gradient clipping to limit the magnitude of the backpropagated gradients to 80  [43]. An L2 vector norm is applied to regularise the weights with a scale of $1\times 10^{-7}$. We use two one-dimensional Gaussian blur kernels with size 7 and standard deviation of 1.

### 3.2. Pose Refinement

For geometric verification, we rely on the radar pose refinement pipeline described in Reference [2,3], which we summarise here. This algorithm reflects the understanding that real, correctly identified landmarks are the same distance apart in any two radar scans.

**Figure 3:**
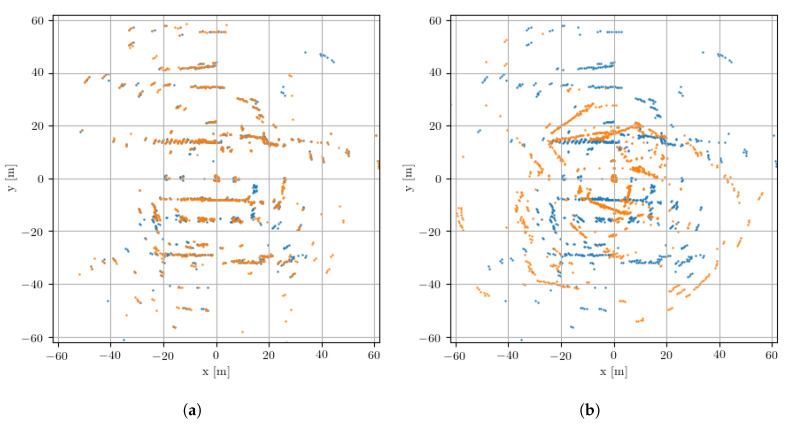
Example landmark sets after detection on radar scans as described in Section 3.2 where in Figure 3a $|L_{1}|=1029$ (orange) and $|L_{2}|=1074$ (blue). In (**a**), the orange landmark set is highly compatible with the blue landmark set. This is because the the scans underlying these sets are taken from very proximal locations. This pair of sets corresponds to the (“bright”) compatibility matrix illustrated in Figure 4b. In contrast, (**b**) shows the same landmark set (orange) alongside a highly incompatible landmark set from a farflung location (blue). This example corresponds to the (“dark”) compatibility matrix illustrated in Figure 4c.

**Landmark extraction:** The landmark extraction algorithm accepts power-range spectra as inputs and returns a set of landmarks, each specified by its range and azimuth. The core idea is to estimate the signal’s noise statistics and then scale the power value at each range by the probability that it corresponds to a real detection. This results in two landmark sets, $L_{1}$ and $L_{2}$, for the consecutive radar scans. Examples of these landmark sets are shown in Figure 3, where $L_{1}$ and $L_{2}$ are shown as the orange and blue sets extracted from (consecutive or non-consecutive) radar scans. These were taken from the *train* portion of the dataset trajectories discussed later in Section 5.

**Motion correction:** It is important for implementation purposes to note that the landmarks we use for localising a query scan to one of the candidate scans in the map are extracted *during mapping*. Therefore, we are able to apply a constant velocity model between motion estimates to reposition landmarks by accounting for motion distortion of an azimuth scan line. This is important considering the relatively low scan rate of a FMCW radar (4 Hz) as compared to the speed of the vehicle (typically 30 $\mathrm{km}h^{-1}$ to 50 $\mathrm{km}h^{-1}$).

**Data association:** The pose refinement algorithm achieves robust point correspondences using high-level information in the radar scan: intuitively, it seeks to find the largest subsets of two pointclouds that share a similar correspondence between pairs of landmarks. First, for every point in the first pointcloud, a potential point match in the second pointcloud is suggested based on a point descriptor that captures the point’s distance and angular information. This results in a set of candidate landmark pairs *B* of size *W*. In Figure 3, the set *B* would be comprised of pairs of landmarks from the orange and blue landmark sets, where *W* would be smaller than or equal to the smaller of $L_{1}$ or $L_{2}$.

**Figure 4:**
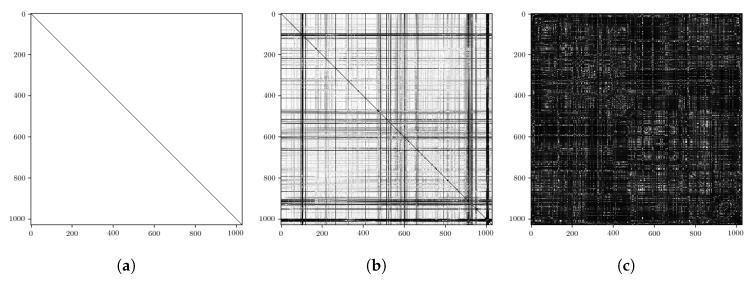
Examples of pairwise compatibility matrices, *C*, which as described in more detail in Section 3.2 are used to solve for the SE(2) motion of the sensor. (**a**) A trivial example arising from the same radar scan passed to the pose refinement module. (**b**) An example using two radar scans in close proximity but with some offset. (**c**) An example where two radar scans are taken from distant locations in the world. From these, it is clear that the “brightness” of the (visualised) compatibility matrix can be used as a quality-score measure for the metric localisation solution as described in Section 4. Importantly, the dimensions of these matrices are determined by the number of matching *landmarks* between the two input radar scans (after data association). Examples of these landmark sets are shown in Figure 3, where in Figure 4b *C* is of shape 1029×1029 corresponding to W=1029 of the $|L_{1}|=1029$ orange and W=1250 of the $|L_{2}|=1074$ blue landmarks in Figure 3a.

**Spectral decomposition:** Non-negative compatibility scores for each pair of these proposed matches are computed and assigned to the elements of a compatibility matrix, *C* of size W×W, in the form of:

(3) $$C_{i,j}=C_{j,i}=\frac{1}{1+|d_{i}-d_{j}|}\in \left(0,1\right],$$

where $d_{i}$ and $d_{j}$ are the distances between a candidate pair *i* and *j*. Examples of these compatibility matrices are shown in Figure 4. We state this here to avoid confusion between the trajectory similarity matrices visualised later (e.g., the groundtruth SE(2) or embedding distance matrices discussed in Section 5 and Section 6).

The vector $m\in {\left\{0,1\right\}}^{W}$ is a solution of the data association problem, where $m_{i}=1$ for a landmark pair match B{i} that is considered or $m_{i}=0$ otherwise. The optimal solution, i.e., the solution that maximises the overall compatibility, can be computed as:

(4) $$m^{*}={\mathrm{argmax}}_{m\in {\left\{0,1\right\}}^{W}}m^{T}Cm.$$

As m is discrete, the above maximisation is computationally difficult. So, in line with Reference [44], we relax this constraint to find the continuously-valued $u^{*}$:

(5) $$u^{*}={\mathrm{argmax}}_{u\in {\left[0,1\right]}^{W}}u^{T}Cu$$

In short, the optimal set of matches maximises the overall compatibility; thus, we use the normalised principal eigenvector of the compatibility matrix. We then apply Singular-Value Decomposition (SVD) to retrieve the registration transformation between the two scans [45].

## 4. Hierarchical Radar Localisation

**Figure 5:**
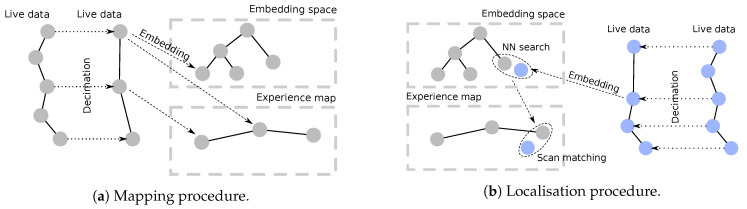
The system is composed of two main phases: mapping (**a**) and localising (**b**). The mapping process entails a two-fold discretisation: one made in a topometric space through an experience map and one made in an embedding space through a kD-tree procedure for fast lookups. Each radar scan in the live data is used to estimate the robot’s motion during both mapping and localisation, but only a portion of them is actually used for localisation. The scans are chosen to be retained through a decimation process, either based on traversed space or time delay.

**Figure 6:**
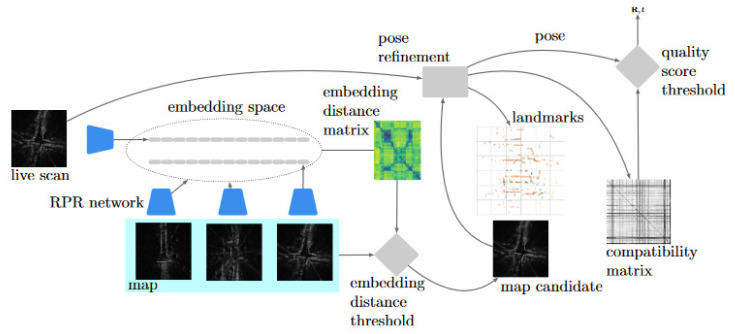
A block diagram illustrating the interaction of the two main submodules in our system: the radar place recognition (RPR) submodule described in Section 3.1 and the pose refinement module described in Section 3.2. A first drive-by of the route, which may be autonomous or piloted manually, is converted into a map of radar scans and their associated RPR embeddings—vectors of size 4096. Likewise, incoming (live) query radar scans are encoded by the feature extraction Convolutional Neural Network (CNN). The query embedding is associated with some subset of the map which is “close” in embedding space. We have illustrated here one such “closest” map frame. This map candidate and the query frame are then passed through the pose refinement module—a process which involves landmark extraction, data association, and rigid-body motion estimation. Importantly, to trade off online and robustness requirements, the two operating thresholds required must be tuned appropriately, as we cover in Section 6.

Figure 5 provides an illustration of the offline and online stages of the proposed system from a perspective of nodes in an experience map (c.f. Section 4.1 below). Both systems described in Section 3 are used in both stages (mapping and localisation), since they are essential in converting the live data flow into the formats used for comparison. The interaction of these two submodules is made more explicit in Figure 6.

In the remainder of this section, we will discuss these steps in more detail.

### 4.1. Mapping

To create a topometric map, our approach is to first decimate the radar scans $s_{i}\in S_{m}$, where $S_{m}$ is the incoming, online sensor stream used for the map creation; the result is a decimated set $S_{m}^{\prime }\subseteq S_{m}$, $S_{m}^{\prime }=\Delta \left(S_{m}\right)$, where Δ(·) is the decimation operation. This step is completely optional and performed with the only purpose of limiting the map size. We approach this task as a combination of two fixed thresholds: the first one in space, $\delta_{m}$, and the second one in time, $\tau_{m}$. We then select a scan $s_{i}\in S_{m}$ iff. $D\left(s_{j},s_{i}\right)\geq \delta_{m}$ and $T\left(s_{j},s_{i}\right)\geq \tau_{m}$—where D(·) or T(·) are the distance operators in space and time, respectively, and $s_{j}\in S_{m}^{\prime }$, $s_{j}≺s_{i}$ is the last selected radar scan.

The selected locations $S_{m}^{\prime }$ and their relative displacements, depicted as nodes and edges, respectively, are then processed to be embedded in the topometric map. The pose refinement subsystem is the main odometry source for the proposed experiments—although our method does not lose generality if we use an external odometry source. To be clear, this odometry source is *not used to propagate localisation candidates* in a Montecarlo Localisation (MCL) or particle-filter incremental fashion. Although we expect that this would bolster performance, this paper is concerned with the integration of the RPR and pose refinement modules, and we leave this to future work.

In this view, the map is composed of two separate digital representations: a so-called *experience map* M and a structured, multidimensional *embedding space* E.

**Experience map:** The experience map addresses the need of the robot to integrate the localisation system to any further planning pipeline and has the task of containing and make available the necessary information. This experience map is illustrated as shaded blue in Figure 6. In our case, the nodes in the experience map will be used down the line for pose refinement. As such, we decided to store both the pointcloud and the point descriptors that we need for pose refinement, sacrificing memory for efficiency and speed further ahead.

**Embedding space:** Secondly, we use a representation of the map for fast retrieval of localisation candidates. To do so, we transform each radar scans into a multidimensional space E by means of the RPR network. This process is illustrated as the blue networks in Figure 6. A nice property of the multidimensional space is that the closest two points in terms of arithmetic difference, the closest in terms of visual similarity. This property lead us to the possibility to structure E into efficient structures for similarity—i.e., closeness—retrieval. We opted for a kD-tree structure due to the implementation simplicity and its deterministic nature, but other possible alternatives exist in literature [46].

### 4.2. Localisation

The mapping procedure is depicted in Figure 5b. Once the map has been created and the AV is traversing the environment again, the localisation procedure can take place. It is possible, as in Reference [47], to perform mapping and localisation in tandem and to create database content as the map is being used for localisation. We leave this for later work (c.f. Section 10).

For now, let us consider an online radar stream $S_{l}$, shown left in Figure 6. As first step, we perform a decimation operation Δ(·) to filter the incoming data, resulting in the set $S_{l}^{\prime }$. Similarly to the mapping procedure, we use a distance and a time thresholds $\delta_{l}$ and $\tau_{l}$ to limit the computational burden of the localisation process.

The localisation pipeline is further composed by three stages. First, the embedding space E is used to retrieve *n* closest matches in terms of visual similarity to the current radar scan, then each of them is tested for geometrical similarity and, if positive, the planar displacement between the pair of scans that best match is computed.

**Visual Similarity:** In this stage, the topological localisation network is used to retrieve from the embedding space *candidates* which are proximal in the multidimensional embedding space to the current radar scan. As mentioned before, we measure closeness with the euclidean distance ${\left||\;\cdot \;|\right|}_{2}$ and query the balanced kD-tree structure used as map for all the candidates within a certain range *E*. This is shown as the embedding distance threshold in Figure 6. *E* is set to limit the search to the nodes with high visual similarity to the online radar scan; nevertheless, since the computational power present on the robot is finite and the map can include many nodes that are visually similar, we can set a maximum number of nodes to be retrieved, *N*. *N* is set to match the temporal performances needed by the system, i.e., the maximum number of scans that can be processed in a useful time period by the final stage of the pipeline.

**Geometrical Similarity:** Once n≤N candidates are retrieved from the embedding-space map (c.f. Online querying in Section 3.1), a verification stage is carried out on each of them. To this end, we perform a geometric verification on the two pointclouds extracted from the online, query radar scan and each of the candidates, one at a time. The choice is to perform the geometric verification as second stage since it is orders of magnitude slower than the visual-similarity search.

Since the method proposed in Reference [2] does not include introspection for assessing the fitness of the relative pose solution and [5] requires training a model with supervision by an external sensor, we opt for a radar-only solution that takes advantage of the compatibility matrix *C* (c.f. Spectral decomposition in Section 3.2). Since *C* is computed starting by scan-dependent descriptors, to increase the speed of the pipeline, it is possible to cache in the map itself the subproducts of the procedure (cf. Experience map in Section 4.1).

In the design of a *quality score* *s* for a pose refinement solution, consider the compatibility matrix *C* of Equation (3). Each compatibility score (for a pair of matching landmarks between consecutive radar scans) is a member of the set (0,1], where a higher value of compatibility score means a higher confidence that the pairs do actually match. In this scenario, a perfect compatibility matrix would contain 1 for every candidate pair (i,j).

In this light, we can design *s* of a match between two radar scans as the average value of the compatibility matrix *C*, i.e.,

(6) $$s=\frac{\sum_{i,j}C_{i,j}}{M^{2}-M}\;\forall i,j\in \left\{1\cdots N\right\}\;|\;i\neq j,$$

where *M* is the dimension of the squared compatibility matrix *C*, defined in Equation (3) on page 7. We subtract the trace of the matrix from the score since it does not contain any information about the geometrical compatibility of the points between the two scans. The quality score *s* can then be compared against a threshold Σ to define if the radar-scan pair is indeed a loop closure. This threshold is shown as the quality score threshold in Figure 6.

This quality score can be interpreted as the “brightness” of the compatibility matrix visualisations shown in Figure 4. The corresponding thresholded quantity in the “ScanContext” hierarchy of Reference [11,35] is computed by finding the most likely rotational alignment between radar scans using cosine-similarities. In contrast to rotation alignment alone, our method exploits a metric on the full 3DoF relative pose of the scans, which is itself a byproduct that “ScanContext” does not provide. Additionally, the “ScanContext” method relies directly on radar power returns, which are prone to sensor artefacts (e.g., multipath reflections), while our measure of quality is computed from the compatibility of landmarks which are themselves detected in a manner which is designed to be robust to these effects (c.f. Section 3.2).

**Pose Computation:** Lastly, once one or more candidates are acknowledged as loop closure—by comparing the value of *s* against the threshold Σ—the final step of the pose-computation pipeline can be carried out to estimate the displacement between the query scan and the candidates—resulting in the pose solution R,t shown top-right in Figure 6.

## 5. Experimental Design

This section describes the experimental setup which is the basis for the results to follow in in Section 6 and Section 7. Specifically, as this work deals with the novel integration (c.f. Section 4) of two (c.f. Section 3) seminal radar techniques, Section 6 spends some time describing the hyperparameter tuning suitable for effective use of the sensor for the application proposed. To this end, we lay out localisation performance and processing requirements in Section 5.2 and Section 5.3. Section 5.1 begins by describing the dataset required for this optimisation, as well as the training and testing of the system as in Section 7.

### 5.1. Dataset

The experiments are performed using data collected from the *Oxford RobotCar* platform [48]. The vehicle, as described in the recently released *Oxford Radar RobotCar Dataset* [49], is fitted with a CTS350-X Navtech FMCW scanning radar.

**Groundtruth Location:** The groundtruth database is curated offline to capture the sets of nodes that are at a maximum distance (15 m, see Section 5.2 below for more detail) from a query frame. This is peformed using the same data structures and operations as the discussion around Equation (2) which deals with online querying of the RPR embedding space—but this time in a metric space measured by an inertial system—as the initial phase of our proposed hierarchical localisation system. This has the added benefit of giving us ground truth rigid-body poses, as well as the topological matches, required to train our CNN. Through this, we create a graph-structured database that yields triplets of nodes for training the representation described in Reference [8] and summarised in Section 3.1.

To this end, we adjust the accompanying groundtruth odometry described in Reference [49] in order to build a database of groundtruth locations. We manually selected a moment during which the vehicle was stationary at a common point and trimmed each ground trace accordingly. We also aligned the ground traces by introducing a modest rotational offset.

**Figure 7:**
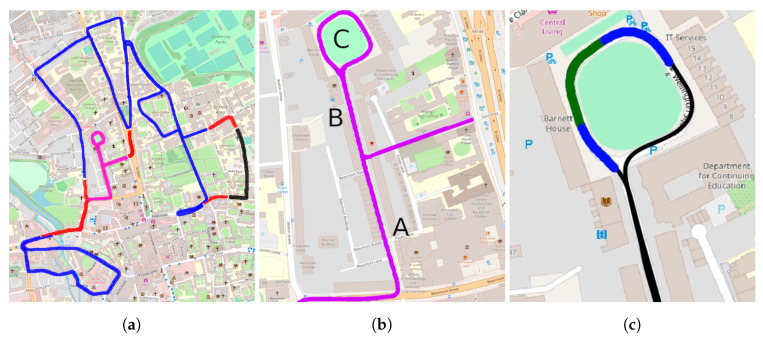
(**a**) *train* (blue), *valid* (black), and *test* (magenta) splits of the full *Oxford Radar RobotCar Dataset*. Red portions serve as padding between splits to account for the long range of the radar. (**b**) Example of *test* split for one of the traversals. A and B are straight portions which look very similar, while C is a very uniform-looking roundabout; these properties are represented in the embedding distance (c.f. Figure 8). Moreover, B is driven twice, in the two opposite directions. The true positive boundary (c.f. Section 5.2) is shown green in (**c**). This 25 m threshold is deemed suitable even in tight roundabouts, such as this, where a larger boundary (blue) would make the location of the vehicle in this tight turn ambiguous.

**Trajectory Demarcation:** As shown in Figure 7a, each approximately 9 km trajectory in the Oxford city centre was divided into three distinct portions: *train*, *valid*, and *test*.

The network is trained with groundtruth topological matches between two reserved trajectories in the *train* split – 2019-01-10-11-46-21-radar-oxford-10k and 2019-01-10-14-50-05-radar-oxford-10k from ori.ox.ac.uk/datasets/radar-robotcar-dataset/datasets.

The *valid* split selected was quite simple, consisting of two straight periods of driving separated by a right turn. This split is simply used to monitor the losses as the RPR module is learning.

The *test* split (c.f. Figure 7b), upon which the results presented in Section 6 to Section 8 are based, was specifically selected to feature vehicle traversals over portions of the route in the opposite direction; data from this split are not seen by the network during training. To be clear, no part of this *test* split—including the portion where streets, lanes, and alleys are driven in reverse—has any overlap whatsoever with the *train* or *valid* split. This is emphasised in Figure 7a.

**Figure 8:**
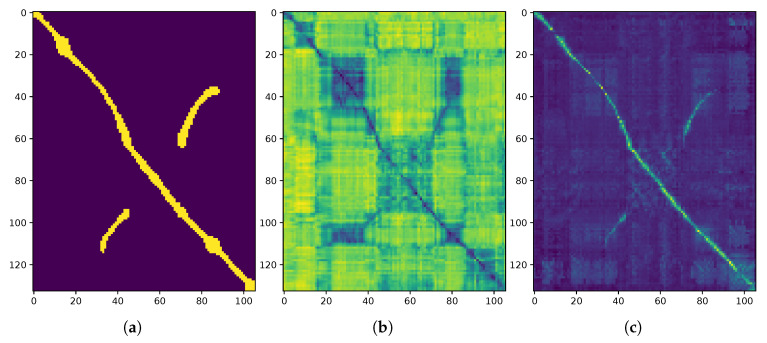
Graphical representation of the SE(2) distance with 25 m threshold applied (c.f. Section 5.2) to form a binary mask used as ground-truth (**a**), embedding distance (**b**) and quality score (**c**) for each pair of radar scans in two separate trials. For each matrix visualised here, the vertical and horizontal axes are in units measuring the number of frames in the query and reference trajectories, respectively. Note that in (**b**) frames which are proximal in embedding space are shaded dark, while the inverse is true for (**c**), where frames with high quality-scores are shaded lightly. It is especially important to note that the quality-score matrix in (**c**) is not computed in its entirety during online operation (prohibitively computationally expensive) but is shown in full here for illustrative purposes.

Portion B of the test set (as shown in Figure 7b) is traversed twice, in opposite directions, as depicted by the off-diagonal positives in Figure 8a. The localisation pipeline can detect the backward traversals, as can be noticed by the low values in embedding distance—Figure 8b—and high quality score—Figure 8c. Furthermore, it is interesting to notice the squared-looking blue portions of the embedding distance: these are portions in the map which are not geometrically contiguous, but are similar nevertheless in embedding space; in particular they are the straight portions of the test set—A and B in Figure 7b—and different sections of the roundabout—C. We additionally observe that the same portions of the map are not as close in quality score, meaning that, even if they look alike, the geometries of the scans do not match.

**Foray Reservation:** We use in total 30 forays from the *Oxford Radar RobotCar Dataset*:

- 2 forays for training,
- 2 forays for hyperparameter tuning,
- 1 foray for mapping, and 
- 25 forays for localisation.

The results focus on a TR scenario, in which all remaining trajectories in the dataset are localised against a map built from the test set of the first trajectory – 2019-01-10-12-32-52-radar-oxford-10k from ori.ox.ac.uk/datasets/radar-robotcar-dataset/datasets – that we did not use for learning or hyperparameter tuning, totalling 25 trajectory pairs (and 26 km of driving) with the same map but a different localisation run.

### 5.2. Localisation Performance Requirements

In the groundtruth SE(2) database, all locations within a 25 m radius of a groundtruth location are considered true positives, whereas those outside are considered true negatives. We consider—due to the long radar sensing horizon on the order of 165 m—that putative matches out to 25 m are well within the capabilities of a downstream radar-only relative pose estimation system as described in Section 3.2. This is further illustrated and discussed in Figure 7c.

As the localisation solution is designed to be used in a software autonomy stack, we consider that false positives are potentially disastrous for motion control and pay heed to recall at 100% precision as the *optimal point* on the precision-recall curves which follow.

For positional errors, we attribute the bias in error (i.e., 10 m) to the manual alignment (c.f. Groundtruth Location in Section 5.1) and focus our discussion instead on either the stability or variance of these errors.

### 5.3. Online Requirements

While $\delta_{m}$ and $\tau_{m}$ (c.f. the mapping phase of our proposed system in Section 4.1) do not influence the online performances of the pipeline—if not in a larger or smaller search pool for candidates—the maximim number of topological candidates *N* and the localisation-stream decimation parameters $\delta_{l}$ and $\tau_{l}$ (c.f. the localisation phase of our proposed system in Section 4.2) are crucial design parameters. This is due to the desire that the pipeline must have a processing rate which is of the same order of magnitude of the live radar stream. Indeed, although the intermediate results needed by the procedure are calculated during the odometry estimation—e.g., pointclouds and point descriptors—the pose refinement pipeline works at about 7 Hz, while the Navtech radar sensor in use has a sampling frequency of 4 Hz.

We dictate as an online requirement that localisation solutions should be reported with $\delta_{l}$ no more than 15 m of linear vehicle motion. This distance serves as it is smaller than the true positive boundary (c.f. Section 5.2). Thus, given a urban vehicle speed limit of 50 km $h^{-1}$, the resulting localisation rate is 0.93 Hz (or a delay of 1.08 s—which we approximate to $\tau_{l}=1$ s—between reported localisation solutions). Under these assumptions, we feed the pose refinement subsystem with the best N=5 candidates from the topological localisation subsystem to address for the kD-tree lookup time. For simplicity, we set $\delta_{m}=\delta_{l}$ and $\tau_{m}=\tau_{l}$ without loss of generality.

Once the five candidates have been geometrically tested, only the candidate which achieved the highest quality score will be used for localisation.

## 6. Hyperparameter Tuning

Here, we reserve two experiences—2019-01-10-14-02-34-radar-oxford-10k and 2019-01-10-15-19-41-radar-oxford-10k from ori.ox.ac.uk/datasets/radar-robotcar-dataset/datasets – for tuning the hyperparameters needed for the system to operate, namely the maximum embedding distance threshold *E* and minimum match quality score Σ to accept as a verified match.

**Figure 9:**
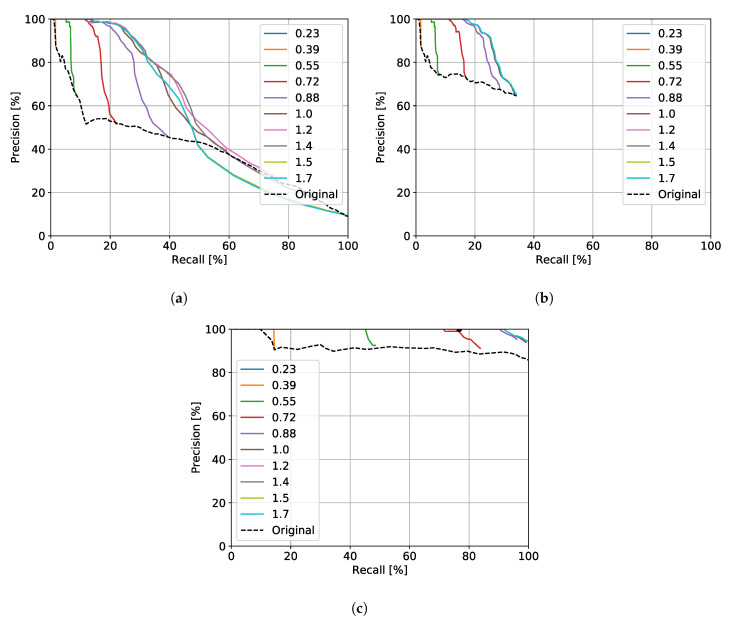
Families of precision and recall (PR) curves for hyperparameters tuning as described in Section 6. These curves represent three different strategies for querying first the visual similarity and then the geometric similarity as measured by Euclidean distance and quality score in the embedding space and pose refinement stages of our pipeline. In (**a**), the legend values show embedding distance thresholds, *E*, below which all candidates for a match to the query frame are measured for their quality score by pose refinement. The parameter which is varied over each curve is the quality score threshold to take as a “correct” match, Σ, from the most (top) to the least (bottom) strict value. “Original” refers to the results reported in Reference [8] with no pose refinement (only RPR), i.e.,  as Σ=0. However, as described in Section 5.3, for online operation, we require that only 5 candidates be passed downstream to the pose refinement submodule; therefore, in (**b**), the 5 closest candidates (also within an embedding distance threshold of *E*) are used to compute quality scores which must be higher than Σ to be considered a match. We select the lowest embedding distance that achieves highest recall at 100% precision, i.e., E=1.0, at a quality score threshold of Σ=0.421; this is based on tuning the system to the “knee” of the PR curve, where we achieve 100% precision. While before we were considering each candidate singularly, in (**c**) we show the performances of the system considering a successful localisation if the best *single* candidate—i.e., with the *best* quality score match from amongst the 5 candidates closest within embedding space—is a positive match. Here, we can achieve much higher recall at 100% precision, but at the cost of sacrificing correctness for all 5 candidates—which may be useful in a multi-particle filtering localisation scheme, for example.

Figure 9 shows the resultant families of precision and recall (PR) curves.

When not including verification by the pose refinement module (c.f. Section 3.2), these PR curves give us a sense for how good the embedding metric space illustrated in Figure 2 learned by the radar place recognition CNN (c.f. Section 3.1) is.

Figure 9a represents the curves where all the queried candidates—with embedding distance lower than the set threshold *E*—are tested for their quality score; Figure 9b, instead, assumes only the five candidates with the lowest embedding distance—also lower than *E*—are tested for scan-matching. We see no relevant difference between the two families, except that the use of only five candidates leads to a lower maximum recall, since many of the candidates are discarded.

Based on a design principle in which we require 100% precision (c.f. Section 5.2) and that online requirements limit the number of scan-match calls to 5 (c.f. Section 5.3) we chose the minimum embedding distance and maximum quality score that lead to the operating point—E=1.0 and Σ=0.421, respectively, which are used in the following experiments. With these parameter values, the improvement in recall at 100% precision with geometric verification is on the order of 15%.

So far, we evaluated each candidate singularly, i.e., in the count of positive and negative matches, each node in the localised and mapped trajectory is taken in consideration as separate entity. In a real localisation task, though, we consider a successful localisation if a queried frame from the localisation trajectory has a match in the map. For this reason, Figure 9c shows the PR curves obtained by using only the best quality-score match between the 5 selected one. we can see how our choices for *E* and Σ in this context are sub-optimal for the task, since it is possible to achieve higher recall either by rising *E* or lowering Σ. In short, we decided not to rise *E* or lower Σ as a further layer of robustness so that all the 5 localisation candidates would be actual loop closures.

We compare our system (from now on referred as kRadar+SM) against two different baselines. First, we use topological matches alone with the same *E* threshold (referred as kRadar@1); this comparison will expose the benefit of the pose refinement pipeline as employed in the second stage of the pipeline. Moreover, for more fair comparison, we use the best match in embedding distance with the highest threshold *E* which leads to 100% precision. The analysis in Figure 9c leads to a value of E=0.36; therefore we refer to this baseline as kRadar@0.36. This comparison will show how the usage of five candidates will greatly increase the recall of the system. Indeed, while the precision remains at 100%, the recall drops from about 75% to about 10%—as in Figure 9c.

## 7. Localisation Performance

In this final results section, we explore an RTR scenario by showing aggregate and metric localisation performance when localising a single experience over a map (c.f. Section 7.1) and then localising the remaining experiences in the month-long dataset against that same map (c.f. Section 7.2).

### 7.1. Localisation of a Single Experience

In this experiment, we evaluate the accuracy of the reported poses over a repeat pass on the one traversal reserved as map – 2019-01-10-12-32-52-radar-oxford-10k from ori.ox.ac.uk/datasets/radar-robotcar-dataset/datasets.

**Figure 10:**
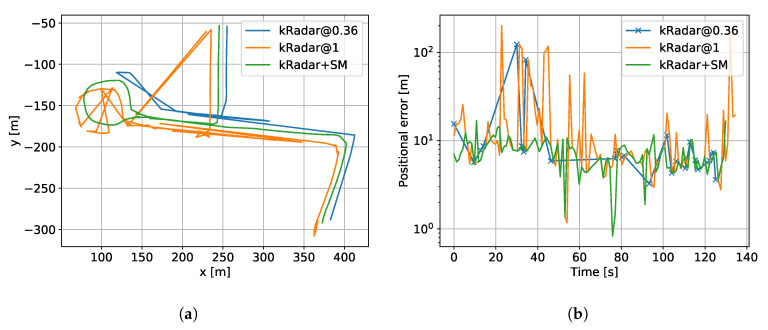
(**a**) Qualitative view of the localisation process. The tracks have been offset for visualisation purposes. (**b**) Positional errors as the localised trajectory one single trial. kRadar@0.36 has been labelled with crosses due to its low recall. Please notice the logarithmic scale.

**Figure 11:**
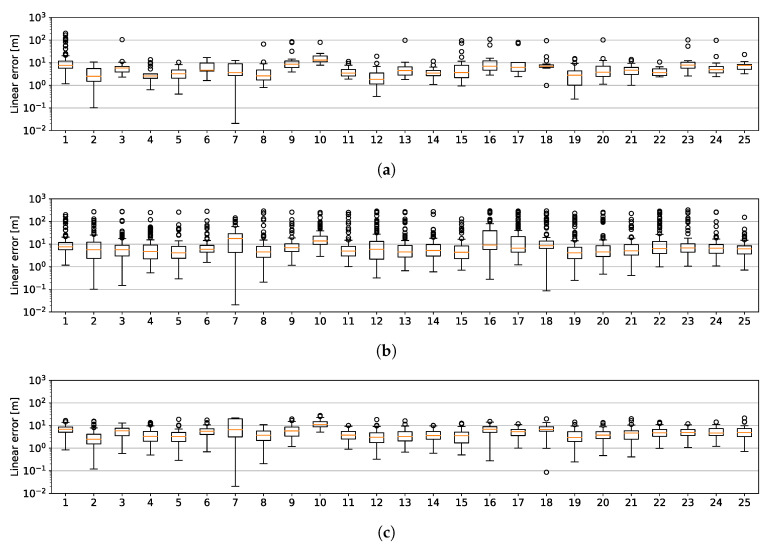
Statistics of the positional error as the localised trajectory is varied over the course of a month and the map is held constant. (**a**) represents kRadar@0.36 (**b**) kRadar@1 and (**c**) kRadar+SM. As described in Section 5.2, there is a bias in these errors (of approximately 10 m) attributed to the manual alignment of the groundtruth odometry traces. Despite this, our proposed system is shown in Figure 11c to exhibit tighter variance over the course of this experiment. Please note the logarithmic scale.

In this experiment, our system achieves 100% precision and 19.7% recall, being able to correct the faulty cases of kRadar@1 which achieves 90% precision and 97.3% recall. From Figure 10a, which shows the qualitative results of projecting the localisation results onto the ground-truth map trajectory, one can notice how most of the errors includes a mismatch between portions A and B, or within C (c.f. Figure 7b). Please note that the trajectories in Figure 10a are offset for *visualisation purposes only* and that these offsets are *not applied* when computing the metric errors reported in Figure 10b, statistics of metric errors reported in Figure 11, or precision-recall performance reported in Figure 9.

Even if tuned for 100% precision, kRadar@0.36 does not achieve it, remaining at 92.3% precision and only 19.7% recall. This result shows that topological localisation solutions alone are hardly enough for a complete system and geometric consistency is an effective way to correct for faulty candidates.

Figure 10b shows the positional deviation of the localisation solutions against the ground-truth position of the localised traversal. This is a further confirm that geometrical compatibility can lead to more robust systems, discarding bad matches even if their embedding distance is lower than the threshold.

### 7.2. Month-Long Localisation

To better confirm the previous results, in this experiment we investigate the utility of a single experience to localisation outlook over many repeat passes. Figure 11 shows the statistics of the positional error as functions of trajectory number—or equivalently, time—using kRadar@0.36 (c.f. Figure 11a), kRadar@1 (c.f. Figure 11b) and our proposed system (c.f. Figure 11c). Here, these performance metrics remain relatively constant throughout the month. We assert that no such guarantee is available in camera- or LiDAR-based TR systems (due to appearance change driven by condition) and attribute any noticeable variation to structural variation and scene dynamics (moving cars or people). Specifically, consider that the upper bound for the lateral error for the first localised trajectory (left-most) in Figure 11c is only on the order of $10^{1}$—minor errors limited to the same street that the car is truly present on—while, for Figure 11a,b, it is on the order of $10^{2}$ to $10^{3}$—grievous errors on the scale of the extent of the dataset. This severe failure of performance is consistent across dataset logs, or localised trajectories, in Figure 11b, and not limited to this first experience as in Figure 11a. Figure 11c, our proposed system, maintains (maximum) errors on the order of $10^{1}$ consistently across the month-long localisation effort.

**Figure 12:**
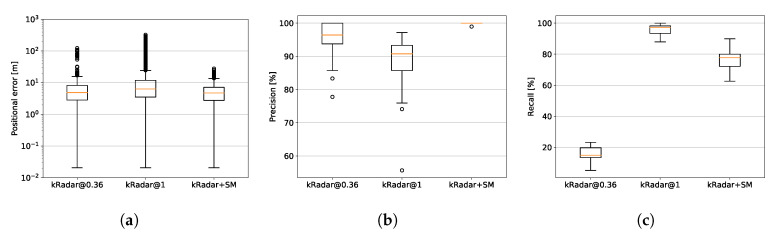
Statistics for positional error (**a**), precision (**b**), and recall (**c**) for the proposed system and the two considered baselines. As described in Section 5.2, there is a bias in these errors (of approximately 10 m) attributed to the manual alignment of the groundtruth odometry traces. Despite this, our proposed system is shown to exhibit tighter variance over the course of this experiment.

As seen also in Figure 12a, in a more compact way, our system exhibits the highest performance in rejecting incorrect matches, thus limiting the positional error. While also the lower threshold in kRadar@0.36 increases the ability in rejecting the bad matches, it never reaches the performances of the complete system. Moreover, Figure 12b,c show how kRadar@0.36 increases precision to the detriment of recall, while kRadar+SM achieved 100% precision—except a single case with 98.96%—with far less effect on recall.

## 8. Benchmark Comparison

In this section, we briefly examine how the contribution compares against other solutions within reach from the broader community.

**Figure 13:**
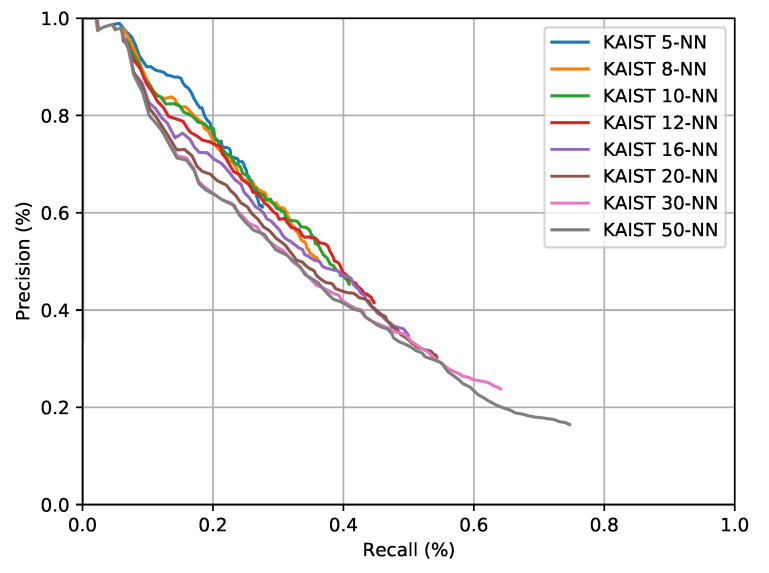
A family of pr curves for the method of Reference [11] as run on the dataset collected in central Oxford.

The solution with the most readily available public implementation is “ScanContext” [35]. This has been adapted to radar in Reference [11]. However, only a C++ implementation for LiDAR is publicly available. Therefore, we have used in generating the results presented in this section our own implementation. Specifically, we use a ring key length of 120 and downsample the radar bins to 40—in the same way as presented in Reference [11,35]. We show, in Figure 13, a family of pr-curves, each of which uses a different number of NNs, starting from 5 and going up to 50—with the later being used to report the results in Reference [11]. These curves are generated for the *test* portion of the *Oxford Radar RobotCar Dataset*, as for the results discussed in Section 6 above.

Considering that the maps (c.f. Section 4.1) for the *test* portion typically contain 150 keyframes (spaced out by vehicle motion), the key takeaway from Figure 13 is that, no matter how many NNs we pass from the place recognition stage to the pose refinement stage of the method presented in Reference [11], high recalls at high precisions are not achieved in the fashion we show for our proposed system in Figure 9c. Consider additionally that the method presented in Reference [11] also only recovers pose up to rotation, rather than the full SE(2) relative pose of the vehicle with respect to the map as is available in our method.

Please also note that the precision-recall performance reported for the “ScanContext” method as applied to radar here is worse than that reported in Reference [11]. We attribute this to our definition of true and false positives and negatives. In Reference [11], a query’s localisation solution is considered correct if the top retrieved candidate is within a certain distance of the actual location of the query. In our work, however, all candidates within that certain distance are considered bonafide matches that must be retrieved.

## 9. Conclusions

We have presented an application of recent advances in learning representations for imagery obtained by radar scan formation for place recognition and seminal work in radar pose estimation to an autonomy-enabling hierarchical localisation process. We demonstrated the efficacy of the proposed system on the largest radar-focused urban autonomy dataset collected to date and showed that the proposed system:

1. can be used to bolster the performance of topological localisation by geometric verification,
2. reports accurate poses in a TR scenario,
3. maintains localisation performance over long time scales, and
4. lends itself well to lifelong navigation techniques for improving localisation.

Specifically, we suggest a range of sensible methods for tuning the system which are suitable for online operation and achieve over the course of a month of localisation trials against a single static map high recalls at high precision and much reduced variance in erroneous metric pose estimation. Depending on the nature of this tuning, we can achieve 100% precision at 85% recall with the single, best match (see Figure 12b,c). This performance is shown to compare favourably against another hierarchical radar localisation process, namely “ScanContext”.

While closed-loop motion control experiments were out of the scope of this paper, we expect that the findings reported here are a strong motivator for exploiting this sensor technology for large-scale mobile robotic navigation in the future, specifically radar teach-and-repeat modes of autonomy.

## 10. Future Work

In the future, we plan to retrain, retune, and test the system on the all-weather platform described in Reference [50] and in off-road scenarios as planned in Reference [51]. We plan to explore experimental settings which place stress on modern techniques for teach-and-repeat (TR) autonomy using cameras, LiDARs and radars. However, even though the place recognition stage is largely sensor agnostic, considering that this work is radar-specific in the pose refinement stage of its hierarchy, this future experimental programme will revolve around adverse conditions themselves.

We expect that the significance of this work will be persistent and anticipate follow-on investigation of efficient methods for selecting which experience to trust for localisation in a multi-experience TR scenario as in Reference [52], as well as predictive systems for characterising localisation performance in TR envelopes around the taught path as in Reference [53].

Finally, we are interested in the density of experiences in a selective recording of memory and expect (due to the immunity of radar to weather and illumination) that fewer memories would be required to represent places than in comparable vision-based systems [47].

## Abbreviations

The following abbreviations are used in this manuscript:

fmcw	Frequency-Modulated Continuous-Wave
rtr	radar teach-and-repeat
cnn	Convolutional Neural Network
vlad	Vector of Locally Aggregated Descriptors
dl	Deep Learning
wsl	Weakly-Supervised Learning
vo	Visual Odometry
gps	Global Positioning System
ro	Radar Odometry
uwb	Ultra Wide Band
fft	Fast Fourier Transform
slam	Simultaneous Localisation and Mapping
mmw	Millimetre-Wave
fcnn	Fully Convolutional Neural Network
lidar	Light Detection and Ranging
nn	nearest neighbour
auc	Area-under-Curve
fov	field-of-view
pr	precision and recall
tr	teach-and-repeat
svd	Singular-Value Decomposition
vtr	visual teach-and-repeat
av	autonomous vehicle
ebn	Experience-based Navigation
vpr	visual place recognition
rpr	radar place recognition
tdof	three degree-of-freedom
mcl	Montecarlo Localisation
